# Detection and characterization of the metabolites of rutaecarpine in rats based on ultra-high-performance liquid chromatography with linear ion trap-Orbitrap mass spectrometer

**DOI:** 10.1080/13880209.2016.1236392

**Published:** 2016-12-07

**Authors:** Wei Cai, Ying Guan, Yang Zhou, Yuwei Wang, Huaiping Ji, Zhihua Liu

**Affiliations:** Department of Pharmacy, Hunan University of Medicine, Huaihua, China

**Keywords:** Rutaecarpine, UHPLC–LTQ-Orbitrap, metabolites, characterization

## Abstract

**Context:** Rutaecarpine is an active indoloquinazoline alkaloid ingredient originating from *Evodia rutaecarpa* (Wu-zhu-yu in Chinese), which possesses a variety of effects. However, its metabolism has not been investigated thoroughly yet.

**Objective:** This study develops a highly sensitive and effective method for detection and characterization of the metabolites of rutaecarpine in Sprague–Dawley (SD) rats.

**Materials and methods:** In this study, an efficient method was developed using ultra-high-performance liquid chromatography coupled with linear ion trap-Orbitrap mass spectrometer (UHPLC–LTQ-Orbitrap MS) to detect the metabolism profile of rutaecarpine in rat plasma. First, a blood sample (1 mL) was withdrawn 2 h after oral administration of rutaecarpine in SD rats (50 mg/kg). Second, the blood was centrifuged at 4000 rpm for 10 min and pretreated by solid-phase extraction method. Third, 2 μL of the plasma was injected into UHPLC–LTQ-Orbitrap MS for analysis. Finally, the metabolites of rutaecarpine were tentatively identified based on accurate mass measurements, fragmentation patterns and chromatographic retention times.

**Results:** A total of 16 metabolites (four new metabolites, viz., dihydroxylation and sulphate conjugation products of rutaecarpine (M8–M11)) as well as parent drug itself, including three phase I and 12 phase II metabolites were detected and identified in rat plasma. Hydroxylation, sulphate conjugation and glucuronidation were confirmed as the primary metabolic pathways for rutaecarpine in rat plasma.

**Discussion and conclusion:** These results provide an insight into the metabolism of rutaecarpine and also can give strong indications on the effective forms of rutaecarpine *in vivo*.

## Introduction

Rutaecarpine is an active indoloquinazoline alkaloid ingredient originating from *Evodia rutaecarpa* (Wu-zhu-yu in Chinese). It has been used extensively as traditional Chinese medicine (TCM) for decades and is officially listed in the Chinese Pharmacopoeia (Chinese Pharmacopoeia Commission 2015). In recent years, despite a lack of knowledge in mechanistic details, pharmacological studies have demonstrated that it possesses antithrombotic, anti-anoxic, hypotensive, anti-inflammatory, uterotonic, thermoregulatory, anti-obesity and vasodilatory effects (Liu & Ho 1999; Kobayashi et al. 2001; Liao et al. 2011; Yu et al. 2013).

Previous pharmacokinetic studies of rutaecarpine indicated that it had a low oral bioavailability, which was attributed to the first-pass metabolism or poor absorption from the gastrointestinal tract (Li 2005). Currently, due to the limitation of analytical techniques, the metabolites of rutaecarpine have not been fully investigated (Ueng et al. 2005, 2006; Jan et al. 2006). For instance, only 16 metabolites in urine and 11 in feces were detected and identified (Lee et al. 2005; Kim et al. 2008), whereas no metabolite was characterized in the plasma. Therefore, it is important to detect and identify its metabolism profile of rutaecarpine in plasma, which can help to further understand the mechanism of action of rutaecarpine.

During the past decade, liquid chromatography/mass spectrometry was the main analytical method for the structural characterization of drug metabolites *in vivo* and *in vitro* (Lin et al. 2012; Szultka et al. 2014). Ultra-high-performance liquid chromatography coupled with high-resolution mass spectrometer (UHPLC–HRMS) such as UHPLC coupled with linear ion trap-Orbitrap mass spectrometer (LTQ-Orbitrap MS) significantly contributed to the characterization of drug metabolites due to its higher separation and resolution capacity in a shorter time (Du et al. 2011; Cai et al. 2015).

In the present study, a total of 16 metabolites (four new) as well as the parent drug itself, including three phase I and 12 phase II metabolites in rat plasma were detected and identified based on accurate mass measurements, fragmentation patterns and chromatographic retention times.

## Material and methods

### Chemicals and reagents

**Figure 1.**
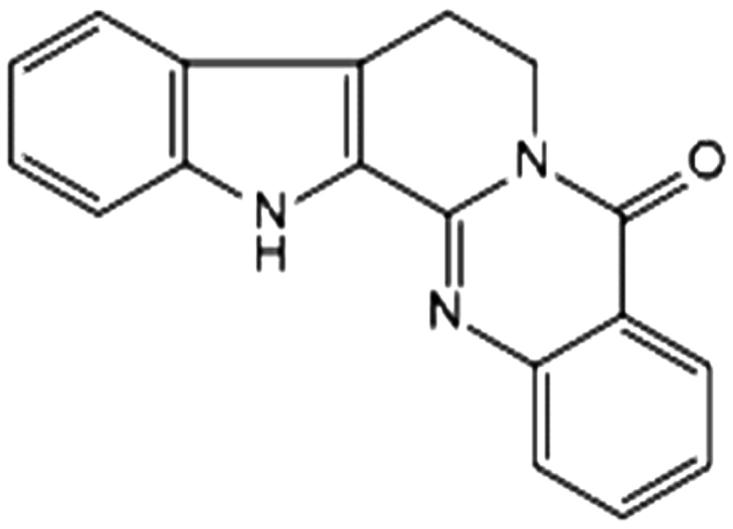
The structure of rutaecarpine.

Rutaecarpine (Figure 1), with a purity of >98% by HPLC analysis was isolated from a 95% ethanol extract of *Euodiae fructus* (identified by professor Wei-feng Dong, Department of Pharmacy, Hunan University of Medicine) in our laboratory. Its structure was identified by comparing UV, MS and NMR data with what is known in the literature. The structure is shown in Figure 1. Grace Pure^TM^ SPE C18 phase extraction cartridges (200 mg/3 mL, 59 μm, 70 Å) were purchased from Grace Davison Discovery Science^TM^ (Deerfield, IL), and all the water used in experiments was purified by a Milli-Q water purification system (Millipore, Billerica, MA). Acetonitrile of HPLC-grade was purchased from Fisher (Fisher, NJ). All other chemicals and regents were of analytical grade and commercially available without further purification.

### Animals and drug administration

Male Sprague–Dawley (SD) rats (250 ± 20 g, Beijing Weitong Lihua Experimental Animals Company, Beijing, China) were acclimatized in controlled environmental conditions (temperature, 24 ± 2 °C; relative humidity, 70 ± 5%) for a week and were allowed free access to food as well as water *ad libitum*. Rats were randomly divided into two groups after a fast of more than 12 h: Group A (*n* = 3); drug group for drug plasma and Group B (*n* = 3); control group for blank plasma. All animal experiments were performed in accordance with the approved animal protocols and guidelines established by medicine ethics review committee for animal experiments of Hunan University of Medicine. Rutaecarpine was suspended in carboxymethylcellulose sodium (CMC-Na) aqueous solution and orally administered to group A at a dose of 50 mg/kg body weight, and an equivalent 0.5% CMC-Na solution was administered by orally gavage to group B. Blood samples (2 mL) were withdrawn in heparinized centrifuge tubes at 2 h following oral administration and centrifuged at 4000 rpm for 10 min to obtain plasma. All plasma samples were stored at −20 °C until further pretreatment and analysis.

### Sample preparations

Plasma samples were pretreated by solid-phase extraction method before UHPLC–MS analysis. An SPE cartridge was pretreated with 5 mL of water, 5 mL of methanol and 5 mL of water, successively. A sample of plasma (1 mL) was processed on a pre-activated solid phase extraction C18 column, and then eluted with 5 mL of water followed by 5 mL of methanol. The methanol eluent was collected and dried under nitrogen gas at room temperature. The residue was re-dissolved in 100 μL of methanol and 2 μL supernatant after centrifugation (12,000 rpm, 30 min at 4 °C) was injected into UHPLC–ESI-LTQ-Orbitrap MS for analysis.

### Instrumentation and conditions

UHPLC–ESI-LTQ-Orbitrap experiments were performed on a Finnigan LTQ/Orbitrap (Thermo Electron, Bremen, Germany) equipped with an Accela 600 pump LC system via an ESI source (Thermo Electron, Bremen, Germany). The LC system was equipped with a binary pump, an auto-sampler and a degasser. The chromatographic separation was achieved on a Waters ACQUITY BEH C18 column (2.1 × 100 mm i.d., 1.7 μm) at room temperature and a flow rate of 0.3 mL/min. A linear gradient elution of solvent water (A) and acetonitrile (B) was applied with the following program: 0–2 min, 5% B; 2–3 min, 5–10% B; 3–25 min, 10–35% B; 25–30 min, 35–80% B; 30–35 min, 80% B; 35–36 min, 80–5% B; 36–40 min, 5% B.

The ESI mass spectra (MS) were acquired in a negative mode by full scan. The MS analyses were performed under optimized conditions, using a spray voltage of 4.0 kV, a capillary voltage of 25 V, a tube lens voltage of 110 V, a curved desolvation line and heat block temperature, an aux gas (nitrogen) flow rate of 5 arb, a sheath gas (nitrogen) flow rate of 30 arb and capillary temperature of 350 °C. The spectra were recorded in the range of *m/z* 100–800 for MS resolution of the Orbitrap mass analyser at 30,000 units. Data-dependent MS/MS scanning was performed to minimize total analytical time as it can trigger fragmentation spectra of target ions. The collision energy for collision induced dissociation (CID) was adjusted to 30% of maximum, and the isolation width of precursor ions was *m/z* 2.0 Da.

## Results and discussion

### Fragmentation pathway of rutaecarpine

**Figure 2.**
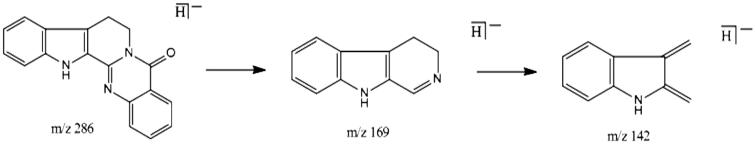
The proposed fragmentation pattern of rutaecarpine.

In order to facilitate the structural identification of metabolites, the MS*^n^* fragmentation pattern of rutaecarpine was investigated in the negative mode detection by ESI (Gao et al. 2012). The parent ion showed a deprotonated ion [M − H]^−^ at *m/z* 286.0982 (2.4 ppm, C_18_H_12_ON_3_). Fragmentation of this parent ion provided a characteristic fragment ion at *m/z* 169.0764 (2.2 ppm, C_11_H_9_N_2_) by the loss of the moiety (C_7_H_3_ON). The fragment ion at *m/z* 142 was produced by the loss of C_7_H_3_ON + CNH from the parent ion, which is useful information in metabolite identification. Besides, the fragment at *m/z* 142 can be formed by the loss of CNH from the ion at *m/z* 169 in the MS^3^ spectra. The proposed fragmentation pattern of rutaecarpine is illustrated in Figure 2.

### Detection and structural elucidation of metabolites

**Figure 3.**
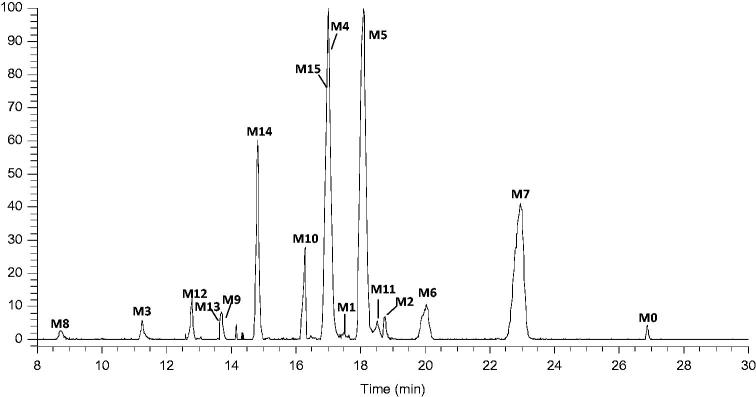
HREIC in 5 ppm for the multiple metabolites in rat plasma *m*/*z* 286.0975, 302.0924, 318.0873, 382.0492, 398.0441 and 478.1245.

After comparison of the high-resolution EIC (HREIC) of drug samples with corresponding control samples, a total of 16 metabolites as well as the parent drug itself were detected and identified. The HREICs of drug samples are shown in Figure 3. The chromatographic and mass spectrometric data of the parent drug and its metabolites are shown in Table 1.

**Table 1.** Summary of rutaecarpine metabolites in rat plasma.

Peak	*t*_R_	Theoretical mass *m/z*	Experimental mass *m/z*	Error (ppm)	Formula [M − H]^−^	MS/MS fragment	Reactions
M0	26.99	286.0975	286.0982	2.4	C_18_H_12_ON_3_	MS^2^ [286]: 169(100), 142(5) MS^3^ [169]: 142(100)	Parent drug
M1	17.64	302.0924	302.0932	2.6	C_18_H_12_O_2_N_3_	MS^2^ [302]: 274(100), 287(75), 169(18)	10-hydroxyrutaecarpine
M2	18.78	302.0924	302.0933	3.0	C_18_H_12_O_2_N_3_	MS^2^ [302]: 287(100), 169(20)	3-hydroxyrutaecarpine
M3	11.26	318.0873	318.0882	2.8	C_18_H_12_O_3_N_3_	MS^2^ [318]: 300(100), 290(40)	3,10-dihydroxyrutaecarpine
M4	17.02	382.0492	382.0501	2.4	C_18_H_12_O_5_N_3_S	MS^2^ [382]: 302(100) MS^3^ [302]: 274(100), 287(77), 260(12), 183(11)	Hydroxylation and sulphate conjugation
M5	18.10	382.0492	382.0505	3.3	C_18_H_12_O_5_N_3_S	MS^2^ [382]: 302(100) MS^3^ [302]: 287(100), 274(18), 183(9)	Hydroxylation and sulphate conjugation
M6	20.04	382.0492	382.0505	3.3	C_18_H_12_O_5_N_3_S	MS^2^ [382]: 302(100) MS^3^ [302]: 274(100), 287(56), 260(40), 169(5)	Hydroxylation and sulphate conjugation
M7	22.95	382.0492	382.0505	3.3	C_18_H_12_O_5_N_3_S	MS^2^ [382]: 302(100) MS^3^ [302]: 259(100), 287(12), 169(8)	Hydroxylation and sulphate conjugation
M8	8.71	398.0441	398.0452	2.7	C_18_H_12_O_6_N_3_S	MS^2^ [398]: 318(100)	Dihydroxylation and sulphate conjugation
M9	13.89	398.0441	398.0450	2.3	C_18_H_12_O_6_N_3_S	MS^2^ [398]: 318(100)	Dihydroxylation and sulphate conjugation
M10	16.30	398.0441	398.0454	3.3	C_18_H_12_O_6_N_3_S	MS^2^ [398]: 318(100)	Dihydroxylation and sulphate conjugation
M11	18.54	398.0441	398.0454	3.3	C_18_H_12_O_6_N_3_S	MS^2^ [398]: 318(100), 147(5)	Dihydroxylation and sulphate conjugation
M12	12.79	478.1245	478.1255	2.1	C_24_H_20_O_8_N_3_	MS^2^ [478]: 302(100), 274(8)	Hydroxylation and glucuronidation
M13	13.71	478.1245	478.1257	2.5	C_24_H_20_O_8_N_3_	MS^2^ [478]: 302(100), 284(10)	Hydroxylation and glucuronidation
M14	14.84	478.1245	478.1256	2.3	C_24_H_20_O_8_N_3_	MS^2^ [478]: 302(100), 274(4)	Hydroxylation and glucuronidation
M15	16.99	478.1245	478.1255	2.1	C_24_H_20_O_8_N_3_	MS^2^ [478]: 302(100), 274(12), 284(6)	Hydroxylation and glucuronidation

#### Metabolite M0

Metabolite M0 was unambiguously identified as rutaecarpine by comparing the retention time and MS with the authentic reference.

#### Metabolites M1, M2, M4, M5, M6 and M7

Metabolites M1 and M2 were eluted at 17.64 and 18.78 min with the quasi-molecular ions of *m/z* 302.0932 (2.6 ppm, C_18_H_12_O_2_N_3_) and *m/z* 302.0933 (3.0 ppm, C_18_H_12_O_2_N_3_), respectively; this was 16 Da more than that of parent drug, suggesting that they were hydroxylated products of rutaecarpine. By comparing the data in the literature (Lee et al. 2005), they were presumed to be 10-hydroxyrutaecarpine and 3-hydroxyrutaecarpine, respectively. Metabolites M4–M7, possessing deprotonated molecular ions [M–H]^–^ at *m/z* 382.0501 (2.4 ppm, C_18_H_12_O_5_N_3_S), *m/z* 382.0505 (3.3 ppm, C_18_H_12_O_5_N_3_S), *m/z* 382.0505 (3.3 ppm, C_18_H_12_O_5_N_3_S) and *m/z* 382.0505 (3.3 ppm, C_18_H_12_O_5_N_3_S), were detected at 17.02, 18.10, 20.04 and 22.95 min, respectively. The fragment ion at *m/z* 302 was produced by the loss of sulphate group (80 Da) from the precursor ion at *m/z* 382. Based on this analysis, they were plausibly deduced as hydroxylation and sulphate conjugation products of rutaecarpine (Lee et al. 2005).

#### Metabolites M3, M8, M9, M10 and M11

M3 was eluted at 12.26 min and produced its [M–H]^–^ ion at *m/z* 318.0882 (2.8 ppm, C_18_H_12_O_3_N_3_), whose molecular weight was 36 Da more than that of M0, indicating that it was a dihydroxylated product of rutaecarpine. Considering that it is much easier to observe hydroxylated occurring at 3 or 10 position of rutaecarpine, therefore M3 was presumed to be 3,10-dihydroxyrutaecarpine. Metabolites M8–M11 eluted at 8.71, 13.89, 16.30 and 18.54 min, generating the deprotonated molecular ions at *m/z* 398.04 (C_18_H_12_O_6_N_3_S), 80 Da more than that of M3. In their MS^2^ spectra, [M–H–SO_3_]^–^ at *m/z* 318 was all observed, suggesting that it could be attributed to sulphate product metabolite of M3. Therefore, they were tentatively identified as dihydroxylation and sulphate conjugation products of rutaecarpine that were new metabolites.

#### Metabolites M12–M15

Metabolites M12–M15 were eluted at 12.79, 13.71, 14.84 and 16.99 min with the quasi-molecular ions of *m/z* 478.1255 (2.1 ppm, C_24_H_20_O_8_N_3_), *m/z* 478.1257 (2.5 ppm, C_24_H_20_O_8_N_3_), *m/z* 478.1256 (2.3 ppm, C_24_H_20_O_8_N_3_) and *m/z* 478.1255 (2.1 ppm, C_24_H_20_O_8_N_3_), respectively; these were 176 Da more than that of hydroxyrutaecarpine. The fragment ions at *m/z* 302 [M − H−C_8_H_8_O_6_]^−^ in the MS^2^ spectra, showed the existence of glucuronide. By investigating the literature data (Lee et al. 2005), they were presumed to be hydroxylation and glucuronide products of rutaecarpine.

### Proposed metabolic pathways of rutaecarpine

**Figure 4.**
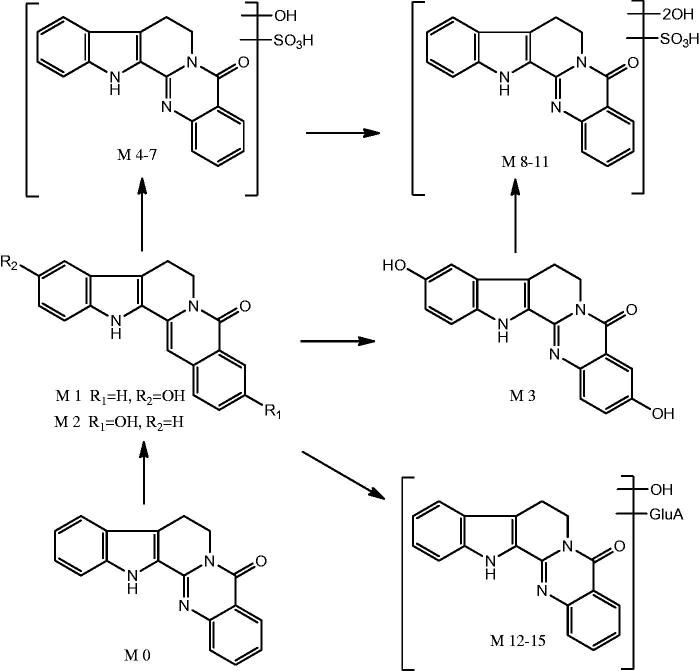
Proposed major metabolic pathways of rutecarpine in rats.

In this study, 16 metabolites (four new) as well as the parent drug were detected in the plasma. The proposed major metabolic pathways of rutaecarpine in the rat plasma are shown in Figure 4. In general, the metabolism of rutaecarpine *in vivo* undergoes hydroxylation metabolic reactions (M1–M2) first, followed by dihydroxylation (M3), and then hydroxylation + sulfate conjugation (M4–M7), dihydroxylation + sulphate conjugation (M8–M11) and finally hydroxylation + glucuronide conjugation (M12) that took place on the position of hydroxyl groups.

## Conclusion

The metabolic profile of rutaecarpine in plasma was investigated following oral administration of a single dose of rutaecarpine to SD rats using a UHPLC–LTQ-Orbitrap MS for analyses. By online LC–MS*^n^* data acquisition and offline data processing methods of the software xcalibur 2.1, a total of 16 metabolites (four new) as well as the parent drug itself, including three phase I and 12 phase II metabolites were detected and identified based on accurate mass measurements, fragmentation patterns and chromatographic retention times. The metabolic reactions of rutaecarpine in rats were hydroxylation, dihydroxylation, hydroxylation + sulphate conjugation, dihydroxylation + sulphate conjugation, and hydroxylation + glucuronidation. In conclusion, profiling the metabolites of rutaecarpine in rats could in future provide comprehensive insights on its pharmacological effects, metabolic fate *in vivo* and effective forms.

## References

[CIT0001] Cai W, Zhang JY, Dong LY, Yin PH, Wang CG, Lu JQ, Zhang HG. 2015 . Identification of the metabolites of Ixerin Z from *Ixeris sonchifolia* Hance in rats by HPLC-LTQ-Orbitrap mass spectrometry. J Pharm Biomed Anal. 107:290–297. 25636166 10.1016/j.jpba.2015.01.004

[CIT0002] Chinese Pharmacopoeia Commission 2015 Pharmacopoeia of the People’s Republic of China. Beijing, China: Medical Science Press; pp 171.

[CIT0003] Du F, Liu T, Liu T, Wang YW, Wan YK, Xing J. 2011 Metabolite identification of triptolide by data-dependent accurate mass spectrometric analysis in combination with online hydrogen/deuterium exchange and multiple data-mining techniques. Rapid Commun Mass Spectrom. 25:3167–3177. 21953973 10.1002/rcm.5211

[CIT0004] Gao P, Wang LZ, Wu RG, Han J, Wang CY, Tang SM, Liu YG. 2012 ESI-ion-trap MS study on fragmentation pathways of evodiamine and rutecarpine. Chin J Pharm Anal. 32:772–774.

[CIT0005] Jan WC, Lin LC, Don MJ, Chen CF, Tsai TH. 2006 Elimination of rutaecarpine and its metabolites in rat feces and urine measured by liquid chromatography. Biomed Chromatogr. 20:1163–1171. 16799925 10.1002/bmc.665

[CIT0006] Kim JH, Lee SK, Seo YM, Choi JH, Shin S, Kang MJ, Kim DH, Jeong HG, Jahng YD, Jeong TC. 2008 Effect of phenobarbital on the pharmacokinetics of rutaecarpine and its metabolite in rats. Biomol Ther. 16:215–218.

[CIT0007] Kobayashi Y, Nakano Y, Kizaki M, Hoshikuma K, Yokoo Y, Kamiya T. 2001 . Capsaicin-like anti-obese activities of evodiamine from fruits of *Evodia rutaecarpa*, a vanilloid receptor agonist. Planta Med. 67:628–633. 11582540 10.1055/s-2001-17353

[CIT0008] Lee SK, Lee DW, Jeon TW, Jin CH, Kim GH, Jun IH, Lee DJ, Kim SI, Kim DH, Jahng Y, et al 2005 Characterization of the phase II metabolites of rutaecarpine in rat by liquid chromatography-electrospray ionization-tandem mass spectrometry. Xenobiotica. 35:1135–1145. 16418066 10.1080/00498250500363742

[CIT0009] Li L. 2005 Metabolic studies of ecodiamine, rutaecarpine and l-tetrahydropalamatine in microbial organisms and rats [Thesis]. Shenyang Pharmaceutical University.

[CIT0010] Liao JF, Chiou WF, Shen YC, Wang GJ, Chen CF. 2011 Anti-inflammatory and anti-infectious effects of *Evodia rutaecarpa* (Wuzhuyu) and its major bioactive components. Chin Med. 6:6–8. 21320305 10.1186/1749-8546-6-6 PMC3046897

[CIT0011] Lin Y, Wu B, Li ZX, Hong T, Chen MC, Tan YZ, Jiang J, Huang CG. 2012 Metabolite identification of myricetin in rats using HPLC coupled with ESI-MS. Chromatographia. 75:655–660.

[CIT0012] Liu ZL, Ho SH. 1999 Bioactivity of the essential oil extracted from *Evodia rutaecarpa* Hook f. et thomas against the grain storage insects, *Sitophilus zeamais* Motsch. and *tribolium castaneum* (Herbst). J Stored Prod Res. 35:317–328.

[CIT0013] Szultka M, Krzeminski R, Jackowski M, Buszewski B. 2014 . Identification of *In vitro* metabolites of amoxicillin in human liver microsomes by LC-ESI/MS. Chromatographia. 77:1027–1035. 25089048 10.1007/s10337-014-2648-2 PMC4111861

[CIT0014] Ueng YF, Don MJ, Jan WC, Wang SY, Ho LK, Chen CF. 2006 Oxidative metabolism of the alkaloid rutaecarpine by human cytochrome P450. Drug Metab Dispos. 34:821–827. 16501007 10.1124/dmd.105.007849

[CIT0015] Ueng YF, Yu HJ, Lee CH, Peng C, Jan WC, Ho LK, Chen CF, Don MJ. 2005 Identification of the microsomal oxidation metabolites of rutaecarpine, a main active alkaloid of the medicinal herb *Evodia rutaecarpa*. J Chromatogr A. 1076:103–109. 15974075 10.1016/j.chroma.2005.04.021

[CIT0016] Yu H, Jin H, Gong W, Wang Z, Liang H. 2013 Pharmacological actions of multi-target-directed evodiamine. Molecules. 18:1826–1843. 23434865 10.3390/molecules18021826 PMC6270287

